# P113 When Young Lupus Patients Do Not Read Textbooks!

**DOI:** 10.1093/rap/rkag113.143

**Published:** 2026-11-03

**Authors:** Rebecca Clough, Elaina Lawrence, Nandita Pai, Kapil Gargh, Kishore Warrier

**Affiliations:** Paediatric Rheumatology, Nottingham University Hospitals NHS Trust, Nottingham, United Kingdom; Paediatric Rheumatology, Nottingham University Hospitals NHS Trust, Nottingham, United Kingdom; Paediatric Rheumatology, Nottingham University Hospitals NHS Trust, Nottingham, United Kingdom; Paediatric Rheumatology, Nottingham University Hospitals NHS Trust, Nottingham, United Kingdom; Paediatric Rheumatology, Nottingham University Hospitals NHS Trust, Nottingham, United Kingdom

## Abstract

**Introduction:**

A 15-year-old South Asian male presented with a 3-month history of isolated periorbital oedema as the first symptom of juvenile systemic lupus erythematosus (jSLE), in addition to then developing unusual complications like splenic infarcts, intractable thrombocytopaenia and Macrophage Activation Syndrome (MAS). This case describes two unique challenges in the diagnosis and management of jSLE. Firstly, the extremely rare initial presentation with isolated eye swelling in the paediatric population. Secondly, the serendipitous diagnosis of an unexpected complication- splenic infarct with no risk factors. Most importantly, this case demonstrates the challenges of diagnosing and managing MAS in a patient with jSLE.

**Case description:**

This patient was a15-year-old boy born in Pakistan and came to the UK at 2 years. Medical history included nose bleeds and chronic persistent urticaria. A paternal half-sister and cousin have SLE.

His presenting symptom was bilateral periorbital oedema unresponsive to antihistamines. Full blood count identified thrombocytopenia, and Idiopathic Thrombocytopenic Purpura was considered. ANA was positive, triggering a Rheumatology referral. The team found that the patient had joint pain, fatigue and easy bruising with no red flags for alternate diagnoses.

Examination revealed symmetrical periorbital oedema with no local erythema, ankle or scrotal oedema. He had erythematous vasculitic spots on his toes. The remaining examination was normal.

Investigations showed immune haemolytic anaemia, thrombocytopaenia, low C3 and C4 with normal ESR and CRP. Autoantibody screen showed strongly positive ANA and double stranded DNA antibodies, with negative triple anti-phospholipid screen. He met the SLICC criteria for the diagnosis of SLE.

Prior to commencing treatment, he was admitted with shortness of breath and haematemesis. Abdominal ultrasound scan suggested splenic infarcts. Echocardiogram showed moderate pericardial effusion. He was given IV Methylprednisolone (IVMP) and started on Hydroxychloroquine. The platelet count dropped to 6 and Intravenous Immunoglobulin (IVIG) was given. Lung function testing demonstrated moderate restrictive pattern with normal HRCT scan.

Given the multi-system involvement and the complications, Cyclophosphamide was chosen for induction but declined by family.

He then spiked fevers without positive microbiology except for weakly positive EBV PCR. CRP and ferritin rose. Differential diagnoses included infection and Macrophage Activation Syndrome (MAS), driven by jSLE or EBV. Despite the absence of validated diagnostic criteria for MAS in SLE, patient met the EULAR criteria for MAS and H scores were rising.

The patient was treated for jSLE primarily (with MAS as a complication of jSLE), with IVMP and IVIG, then Rituximab and Cyclophosphamide. He made excellent recovery.

**Discussion:**

This unique case describes a series of rare features occurring in jSLE - the extremely rare initial presenting symptom of isolated periorbital swelling, the rare complication of splenic infarct in the absence of risk factors, and challenges in the diagnosis and management of MAS occurring in a patient with jSLE.

Differentials for the isolated periorbital swelling include allergy, nephrotic syndrome, mediastinal obstruction and juvenile dermatomyositis but were clinically ruled out. Features of cutaneous involvement, seen in the majority of jSLE patients with periorbital swelling, were absent. Isolated periorbital oedema with no skin changes or nephrotic syndrome as the presenting feature of lupus has only been reported in a small number of paediatric cases. Interestingly, some of these patients had pericardial effusion, as in this case.

Clinical involvement of the spleen in jSLE can include splenomegaly, spontaneous splenic rupture and rarely splenic infarcts, the reasons for which are thought to be anti-phospholipid antibody syndrome (not present in this patient) or vasculitis (probable here).

It can be challenging to diagnose and treat MAS in jSLE. Pancytopenia could be secondary to haematological manifestation of jSLE like immune haemolytic anaemia and thrombocytopenia but are also part of the diagnostic criteria of MAS. The same applies to coagulopathy, abnormal liver enzymes and central nervous system involvement. This highlights the need to have a validated classification criteria for the diagnosis of MAS in jSLE.

Determining appropriate treatment in a patient with a diagnosis of both jSLE and MAS can be challenging too–deciding whether to treat active lupus (Rituximab, Cyclophosphamide) as the underlying cause driving the MAS or to treat MAS (using Anakinra or HLH protocol) and jSLE separately. The family declining Cyclophosphamide initially added to the complexity, as using Rituximab alone could have led to a bigger steroid burden for a longer period.

**Key learning points:**

1. A rare presentation of jSLE with bilateral isolated periorbital oedema without associated skin changes and no clinical features to suggest nephrotic syndrome is unique.

2. The management of intractable thrombocytopenia in this case was challenging, complicated by the onset of MAS. The initial recovery of the platelet count following the first dose of IVIG was offset by the evolution of MAS, which pushed the counts further down, which prompted further IVIG, which works for both ITP and MAS.

3. Although the spleen can be rarely involved in jSLE, splenic infarcts are extremely rare in jSLE and may occur secondary to anti-phospholipid antibody syndrome, which this patient did not have, or presumed vasculitis.

4. There is diagnostic overlap between features of jSLE and MAS, which makes the diagnosis challenging using conventional diagnostic criteria for Haemophagocytic lymphohistiocytosis (HLH) or EULAR criteria for MAS. In the absence of a validated diagnostic criteria for MAS in jSLE, the trend in the investigations is more important than the absolute values and rising probability using H score could be a useful added tool, as quick decision making improves outcomes.

5. When treating MAS in the context of jSLE, high dose systemic corticosteroids are crucial, which work for both. However, consideration is needed for the choice of further steroid sparing treatment (treating jSLE as the driver of MAS or treating MAS separately to jSLE). Most agents available to induce remission in jSLE (Cyclophosphamide, Rituximab) may take weeks to start working, which may leave the patients with higher steroid burden for longer.

6. This case also highlights the importance of ensuring that the treatment is acceptable to the patient and their family, for which honest and clear communication at all stages is crucial and here led to the family agreeing to the use of Cyclophosphamide subsequently.

